# Suicide and Sleep Disorders: The Role of Insomnia, Nightmares, and Circadian Disruption

**DOI:** 10.7759/cureus.112319

**Published:** 2026-07-09

**Authors:** Omofolarin Debellotte, Amuksha Ravindra Shetty, Yuzhu Wang, Ayesha Amin, Vikdeep Mann, Morolayo Fadayomi, Pranati Gudipati, Haidy Hanna, Libina Karippaparambil, Manju Rai

**Affiliations:** 1 Internal Medicine, New York University, New York City, USA; 2 Psychiatry and Behavioral Sciences, Dr. Balabhai Nanavati Hospital, Mumbai, IND; 3 Psychiatry, School of Medicine, St. George’s University, St. George's, GRD; 4 Psychiatry, Fatima Jinnah Medical University, Lahore, PAK; 5 Psychiatry, Brunel University of London, London, GBR; 6 Psychiatry, School of Medicine, St. George’s University, St. George’s, GRD; 7 Psychiatry and Behavioral Sciences, Jawaharlal Nehru Medical College, Belgaum, IND; 8 Psychiatry, Red Deer Regional Hospital Centre, Red Deer, CAN; 9 Psychiatry, New Cross Hospital, The Royal Wolverhampton NHS Trust, Wolverhampton, GBR; 10 Biotechnology, Shri Venkateshwara University, Gajraula, IND

**Keywords:** chronotherapy, circadian rhythm disruption, cognitive behavioral therapy for insomnia, emotional dysregulation, insomnia, neurobiological mechanisms, nightmares, sleep disorders, suicidal ideation, suicide prevention

## Abstract

Suicide remains a leading global cause of preventable mortality, with sleep disturbances, particularly insomnia, nightmares, and circadian disruption, emerging as critical yet underrecognized contributors to suicidal ideation and behavior. This narrative review synthesizes current evidence on the multifaceted links between sleep disorders and suicidality, integrating findings from epidemiological, neurobiological, and interventional studies. Insomnia contributes to suicide risk through pathways of hyperarousal, hopelessness, and impaired emotion regulation, while nightmares are associated with increased risk of suicidal ideation and attempts, potentially exerting an effect distinct from other sleep disorders. Circadian misalignment, including shift work and evening chronotype, further exacerbates vulnerability through neuroendocrine and molecular alterations involving the hypothalamic-pituitary-adrenal axis, serotonergic imbalance, and disrupted clock gene expression. Shared biological mechanisms, such as inflammation, hypothalamic-pituitary-adrenal (HPA) dysregulation, and serotonergic depletion, intersect with cognitive-emotional deficits, compounding risk. Clinically, interventions such as cognitive behavioral therapy for insomnia (CBT-I), imagery rehearsal therapy, and chronotherapeutic approaches have demonstrated efficacy in improving sleep continuity and mitigating suicidality. Routine assessment of sleep quality and circadian patterns should be integrated into suicide risk evaluation across psychiatric and primary care settings. Future research incorporating objective sleep measures, biomarker profiling, and digital monitoring platforms may enable precision risk stratification and early intervention. However, the current evidence base is limited by heterogeneity in study designs, the predominance of observational and cross-sectional studies, and potential confounding from psychiatric comorbidities, warranting cautious interpretation of causal relationships. By addressing sleep as a core modifiable risk factor, clinicians can move toward a holistic, biologically informed, and preventive model of suicide care.

## Introduction and background

Suicide has been identified by the World Health Organization (WHO) as a global public health priority, given its persistently rising incidence despite expanding prevention efforts. It remains the 10th leading cause of death worldwide and the second leading cause among adolescents, imposing a considerable burden on individuals, families, and healthcare systems, with estimated medical and quality-of-life costs approaching USD 490 billion annually [1]. According to 2022 Centers for Disease Control and Prevention (CDC) data, approximately 50,000 suicide deaths occurred in the United States, with suicide ranking among the top four leading causes of death among individuals aged 10-34 years [2]. Suicidal ideation (SI) represents the earliest and most significant precursor to suicidal behavior, underscoring the need for early identification and targeted prevention. While psychiatric disorders such as depression and substance use have long been recognized as major risk factors, emerging evidence highlights sleep disturbances, particularly insomnia and nightmares, as independent and modifiable predictors of suicidality [1-5].

Insomnia, as defined in the Diagnostic and Statistical Manual of Mental Disorders, Fifth Edition (DSM-5), involves persistent difficulty initiating or maintaining sleep, resulting in impaired daytime functioning [1]. Among adolescents, insomnia is associated with diminished cognitive performance, emotional dysregulation, and lowered stress tolerance, factors that collectively exacerbate hopelessness and SI [1,4,5]. A bidirectional relationship exists between insomnia and depression, wherein sleep disruption worsens affective instability, and mood dysregulation further disrupts sleep continuity [5]. Neurobiological evidence implicates prefrontal cortex hypoactivity and serotonergic and dopaminergic imbalances in mediating the link between poor sleep and impaired impulse control, thereby increasing vulnerability to self-harm and substance misuse [1,5].

Nightmares, defined as distressing dreams occurring during rapid eye movement (REM) sleep, have also been associated with an increased risk of suicidal ideation and behavior, potentially exerting an effect distinct from other sleep disturbances while interacting with coexisting psychiatric conditions [1]. Individuals reporting occasional nightmares had an approximately 1.57-fold higher risk of suicide, whereas those experiencing frequent nightmares had an approximately 2.07-fold higher risk compared with individuals without nightmares [1]. Furthermore, nightmares mediate the relationship between insomnia and suicidal ideation, suggesting a shared pathophysiological pathway involving emotional dysregulation and hyperarousal [1].

Beyond insomnia and nightmares, circadian rhythm disruption has emerged as an important contributor to suicide risk. Circadian misalignment resulting from factors such as evening chronotype, shift work, irregular sleep-wake schedules, and altered light exposure has been associated with emotional dysregulation, impaired stress responses, and increased vulnerability to suicidal ideation and behavior [6]. Growing evidence suggests that disturbances in circadian timing influence neuroendocrine signaling, serotonergic neurotransmission, and clock gene expression, providing biologically plausible mechanisms linking disrupted circadian rhythms with suicidality [7]. Recognizing circadian disruption as a modifiable risk factor broadens the understanding of sleep-related pathways to suicide and highlights additional opportunities for prevention and targeted intervention.

Despite growing recognition of sleep disorders as modifiable risk factors for suicidality, the underlying neurobiological and circadian mechanisms remain poorly understood. This narrative review synthesizes current evidence on the roles of insomnia, nightmares, and circadian disruption in SI and behavior, elucidates potential neurobiological and psychosocial pathways, and discusses implications for clinical risk assessment and targeted intervention.

## Review

Methodology

This narrative review was conducted to synthesize and critically appraise current evidence regarding the associations between sleep disorders, specifically insomnia, nightmares, and circadian rhythm disruption, and suicidal ideation and behavior. A narrative approach was chosen because the available literature encompasses heterogeneous epidemiological, neurobiological, clinical, and interventional evidence that is not readily amenable to systematic quantitative synthesis.

Search Strategy

A comprehensive literature search was conducted across the PubMed/MEDLINE, PsycINFO, Scopus, and Google Scholar databases, covering publications from January 2009 to April 2026. The following Medical Subject Headings (MeSH) terms and keywords were used in various combinations: suicide, suicidal ideation, suicide attempt, self-harm, insomnia, sleep disturbance, sleep disorder, nightmares, circadian rhythm, circadian disruption, chronotype, shift work, sleep quality, cognitive behavioral therapy for insomnia (CBT-I), cognitive behavioral therapy for insomnia, imagery rehearsal therapy, chronotherapy, HPA axis, serotonin, neuroinflammation, hopelessness, and emotional dysregulation. Boolean operators were used to construct the search strategy, with OR used to combine synonymous terms within each concept and AND used to combine the major concepts (suicidality, sleep disorders, and therapeutic or mechanistic terms). Search strategies were adapted to the syntax and indexing system of each database. A representative search strategy used for PubMed/MEDLINE was as follows: (("suicide" OR "suicidal ideation" OR "suicide attempt" OR "self-harm") AND ("insomnia" OR "sleep disturbance" OR "sleep disorder" OR "nightmares" OR "circadian rhythm" OR "circadian disruption" OR "chronotype" OR "shift work") AND ("CBT-I" OR "cognitive behavioral therapy for insomnia" OR "imagery rehearsal therapy" OR "chronotherapy" OR "HPA axis" OR "serotonin" OR "neuroinflammation" OR "hopelessness" OR "emotional dysregulation")).

Inclusion and Exclusion Criteria

Studies were included if they reported associations between one or more sleep disorders, including insomnia, nightmares, or circadian disruption, and suicidality outcomes such as suicidal ideation, plans, attempts, or deaths. Eligible studies were published in peer-reviewed English-language journals and involved adult or adolescent human populations. Original research articles, randomized controlled trials, observational studies, prospective cohort studies, systematic reviews, and meta-analyses were considered for inclusion. In addition, one experimental animal study was included because it provided mechanistic insights into the effects of circadian disruption on melatonin regulation and neurobehavioral pathways relevant to suicidality that could not be directly investigated in human studies. Animal studies were considered eligible only if they addressed biological mechanisms with clear translational relevance to the relationship between sleep disturbances and suicide risk and were used solely to support the mechanistic discussion rather than the clinical evidence synthesis.

Study Selection and Data Synthesis

Title and abstract screening were performed independently by multiple authors to assess eligibility. Relevant full-text articles were subsequently retrieved and reviewed. Search results from all databases were exported into Zotero Reference Manager (Falls Church, VA: Corporation for Digital Scholarship), where duplicate records were identified using Zotero's built-in duplicate detection feature and subsequently verified manually by comparing article titles, author names, publication year, journal, and digital object identifiers (DOIs). Duplicate records were removed prior to title and abstract screening. Any disagreements regarding study eligibility were resolved through discussion and consensus among the reviewers, with consultation from the senior author when necessary.

Studies were organized into the following four primary thematic domains: the general relationship between sleep and suicide, insomnia and suicide risk, nightmares and suicide risk, and circadian disruption and suicide risk. Additional themes, including shared neurobiological and cognitive-emotional mechanisms, psychiatric comorbidities, clinical and therapeutic implications, and future research directions, were also synthesized to provide a comprehensive overview of the current evidence.

**Figure 1:**
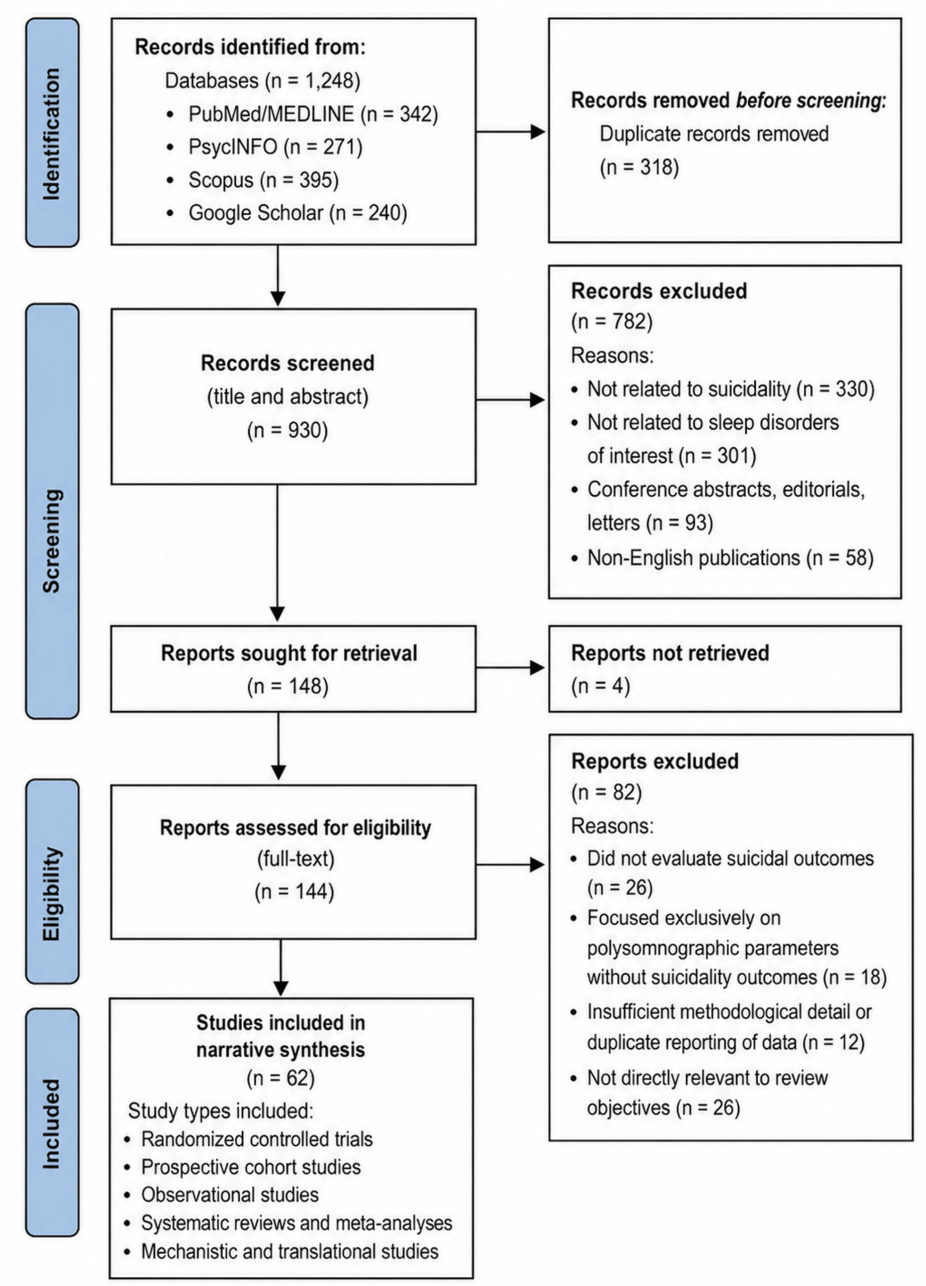
Flowchart of literature selection.

To enhance transparency and reproducibility, a structured literature search and study selection process was undertaken and is illustrated in Figure 1. However, this review was designed as a narrative synthesis rather than a formal systematic review. Consequently, a standardized risk-of-bias assessment or formal methodological quality appraisal (e.g., Cochrane Risk of Bias tool or Newcastle-Ottawa Scale) was not performed. Nevertheless, study quality was considered qualitatively during evidence synthesis by evaluating study design, sample size, methodological rigor, consistency of findings with the broader literature, relevance to the review objectives, and the appropriateness of statistical analyses where applicable. Greater emphasis was placed on evidence derived from randomized controlled trials, longitudinal cohort studies, systematic reviews, meta-analyses, and well-conducted mechanistic investigations. Findings from smaller observational studies, cross-sectional studies, narrative reviews, or studies with important methodological limitations were interpreted cautiously and used primarily to provide supportive or hypothesis-generating evidence rather than as the principal basis for the conclusions. Final interpretations were based on the overall consistency and strength of evidence across multiple studies rather than on individual reports.

Ethical Considerations

As this review is based entirely on previously published, publicly available literature, no ethical approval or informed consent was required. No primary data were collected or analyzed. All cited sources have been appropriately referenced in accordance with standard academic practice.

Suicide and sleep: an overview

Sleep-related factors, including insomnia, nightmares, and circadian irregularities, have emerged as consistent correlates of suicidal ideation and behavior. Meta-analytic evidence in adolescents shows that sleep problems are linked to more than twofold higher odds of suicidal ideation and threefold higher odds of suicide attempts, underscoring sleep as a salient, potentially modifiable risk factor in youth [6]. Prospective cohort work in the Adolescent Brain Cognitive Development (ABCD) Study further indicates that preadolescent sleep disturbance (notably nightmares and excessive daytime sleepiness) predicts new-onset suicidal ideation (SI) and attempts two years later, even after adjustment for anxiety and depression symptoms and family-context covariates [7]. Short-term, intensive monitoring studies complement these findings as follows: worsening sleep across days predicts next-week SI among high-risk youth in treatment [8], and night-to-day designs show that fragmented or poor sleep predicts next-day increases in suicidal thoughts, partly via heightened hopelessness [9,10]. At the intervention level, randomized and translational trials suggest that remission of insomnia with cognitive behavioral therapy for insomnia (CBT-I), including digital delivery, can alleviate and even prevent suicidal ideation, supporting sleep as a tractable prevention target [11].

Multiple biological and psychosocial pathways plausibly connect sleep disruption to suicidality. Sleep-circadian dysregulation is a transdiagnostic contributor to psychopathology, with converging evidence for alterations across homeostatic sleep processes, circadian timing systems, and their neural interfaces [12]. Emerging biological evidence implicates inflammatory and stress-related pathways linking sleep and suicidality, which are discussed in detail later. Psychologically, sleep loss and fragmentation can intensify hopelessness and diminish affective resilience, while impairing executive control and problem-solving, processes central to ideation-to-action models of suicide. Ecological momentary assessment studies show that subjective sleep deficits and objective sleep fragmentation predict next-day suicidal ideation, with hopelessness mediating part of this association [13]. Nightmares may contribute additional risk through conditioned fear, heightened nocturnal arousal, and sleep fragmentation that further erodes emotion regulation [6,7,10].

Circadian and social timing factors add context. Occupational exposures that destabilize sleep-wake timing, long working hours, and shift work are associated with higher odds of suicidal ideation in pooled analyses, consistent with the view that circadian misalignment and irregular schedules can amplify risk, particularly when layered onto existing vulnerabilities [14]. These epidemiological signals align with mechanistic accounts positing that circadian disruption alters reward, stress, and threat circuits relevant to suicidal thinking [12].

Insomnia and suicide risk

Insomnia represents a significant but underrecognized contributor to suicide risk. Over the past two decades, suicide rates have risen substantially, particularly among adolescents, young adults, and middle-aged individuals [15]. Insomnia is highly prevalent in psychiatric inpatient and primary care settings and commonly co-occurs with major depressive disorder (MDD) and post-traumatic stress disorder (PTSD), both established risk factors for suicide [16,17]. McCall et al. further observed that among patients with insomnia, men were more likely than women to attempt suicide, underscoring potential sex-specific vulnerabilities [17]. These findings highlight insomnia as a clinically meaningful and modifiable risk factor that warrants systematic inclusion in suicide risk assessment and prevention frameworks.

An expanding body of randomized trials has examined whether treating insomnia reduces suicidal ideation. The Reducing Suicidal Ideation Through Insomnia Treatment (REST-IT) trial evaluated 103 adults with MDD and active suicidal ideation who received zolpidem-CR combined with a selective serotonin reuptake inhibitor (SSRI) versus placebo plus SSRI [17]. Although improvements in suicidal ideation were non-significant on the Scale for Suicidal Ideation (SSI), reductions were significant on the Columbia-Suicide Severity Rating Scale (C-SSRS), suggesting a partial suicidolytic effect mediated through improved sleep continuity. In primary care, brief CBT-I (bCBT-I) for patients with comorbid insomnia and suicidal ideation improved insomnia symptoms but did not yield statistically significant reductions in suicidal thoughts, indicating that sleep improvement alone may not suffice for all patients [15].

Digital and remote CBT-I programs have broadened accessibility. The Sleep Healthy Using the Internet (SHUTi) platform, tested among U.S. veterans, significantly reduced insomnia severity and hopelessness, though its effects on suicidal ideation were modest [18]. In a large community sample of Australian adults, SHUTi improved sleep quality, depression, anxiety, and suicidal thoughts compared with an attention-control program, demonstrating that digital CBT-I can benefit multiple domains of mental health [19]. Similarly, the Yara smartphone application (Urmia, Iran: Urmia University of Medical Sciences), which incorporates behavioral and relaxation modules for patients with MDD, improved both anxiety and sleep quality but did not significantly affect suicidal ideation [20].

Across these trials, a consistent pattern emerges as follows: interventions targeting insomnia reliably alleviate sleep disturbance and depressive symptoms, but effects on suicidal ideation are secondary and often modest. This indicates that while sleep improvement may indirectly mitigate suicide risk, additional mechanisms, such as cognitive restructuring, emotional regulation, and social connectedness, may be necessary for durable suicidolytic outcomes.

Insomnia contributes to suicidality through overlapping biological and psychological pathways involving hyperarousal, hopelessness, and emotional dysregulation. Physiological hyperarousal, marked by increased cortisol levels, accelerated EEG activity, and elevated metabolic rate, impairs prefrontal inhibitory control and emotional regulation, fostering impulsivity and despair [21]. Hopelessness, one of the strongest proximal risk factors for suicide, often arises from chronic insomnia due to sustained cognitive and emotional fatigue. Individuals experiencing prolonged sleep disturbance may develop rigid, pessimistic thinking patterns, such as “I’ll never get better,” that amplify suicidal cognition. Concurrently, insomnia disrupts rapid eye movement (REM) sleep, a critical phase for emotional processing and adaptive coping. REM fragmentation impairs affective regulation, increasing vulnerability to maladaptive emotional responses [22]. Evidence from the REST-IT trial supports this interplay, showing that as insomnia improved, emotional stability increased [17]. Together, these mechanisms contribute to increased vulnerability to suicidal ideation through interacting biological and cognitive pathways. The shared mechanisms linking sleep disturbances and suicidality are discussed in greater detail in the section "shared mechanisms underlying sleep disorders and suicide."

Given the strong association between insomnia and suicidality, clinicians should incorporate systematic insomnia screening into suicide risk assessments across primary care, emergency, and psychiatric settings. Pharmacological treatments, such as short-term zolpidem-CR, may be used during the acute phase of management, but the risks of long-term dependence and tolerance must be considered [12]. Cognitive behavioral therapy for insomnia (CBT-I), delivered through in-person or digital platforms, remains the first-line, evidence-based intervention for sleep improvement and may indirectly reduce suicide risk. However, remission of insomnia does not necessarily ensure resolution of suicidality, emphasizing the need for multimodal, sustained interventions that integrate sleep management with psychotherapeutic and pharmacological strategies targeting mood regulation, impulsivity, and hopelessness.

Nightmares and suicide risk

Nightmares are recurrent, dysphoric dreams that typically occur during rapid eye movement sleep and are often followed by awakenings and marked distress. Beyond their association with psychiatric illness, convergent evidence indicates that nightmares constitute an independent risk factor for suicidal thoughts and behaviors. Meta-analytic summaries report small-to-moderate effects, with nightmare frequency prospectively predicting suicidal ideation (approximately d ≈ 0.40) and suicide attempts (approximately d ≈ 0.64) even after adjustment for depression, PTSD, and insomnia, supporting an effect that is not solely attributable to comorbidity [23,24].

Although nightmares frequently co-occur with PTSD and depression, growing evidence suggests they exert an independent effect on suicidality, likely mediated by shared mechanisms such as emotional dysregulation and disrupted sleep continuity. In PTSD, trauma-related nightmares may perpetuate core symptoms via re-experiencing and nocturnal hyperarousal, thereby sustaining daytime distress and elevating suicidal risk [24]. Interventions that specifically target nightmares, most prominently imagery rehearsal therapy, reduce nightmare frequency and distress and have been associated with reductions in suicidal thoughts, implying a direct, modifiable pathway from nightmares to suicidality [24]. In depression, recurrent nightmares are linked to persistent negative affect and hopelessness; longitudinal adolescent data indicate that baseline nightmare frequency and nightmare-related distress predict subsequent suicidal ideation, plans, and attempts, with depressive symptoms partially mediating the association [25]. Pooled analyses further suggest that individuals with frequent nightmares demonstrate 1.5- to threefold higher risk of suicidal ideation or behaviors than those without nightmares [26]. Early and cumulative trauma confer additional vulnerability as follows: developmental adversity is associated with heightened autonomic reactivity and recurrent nightmares, which in turn amplify shame, guilt, and hyperarousal-affective states consistently tied to suicidality [27].

Mechanistically, nightmares may heighten suicide vulnerability through interlocking pathways of impaired distress tolerance, conditioned fear/hyperarousal, sleep fragmentation, and maladaptive cognition. Repeated exposure to intensely negative dream affect erodes emotion regulation capacity, lowering distress tolerance and fostering hopelessness and impulsive responding during wakefulness [28]. Classical fear conditioning links internal cues and dream imagery to autonomic activation; persistent sympathetic arousal then generalizes to daytime hypervigilance and intrusive mentation, sustaining suicidal cognitions in vulnerable individuals [28]. Frequent nocturnal awakenings fragment sleep, disrupting processes critical for emotional memory consolidation, mood regulation, and executive control; these changes increase negative bias and impair problem-solving when crises arise [29]. Finally, rumination and repetitive negative thinking appear to mediate part of the nightmare-suicidality pathway, creating self-perpetuating loops in which distressing dreams trigger perseverative cognition that, in turn, predicts further nightmares and increased suicide risk [30]. Together, these pathways highlight how nightmares may contribute to suicidal vulnerability through overlapping emotional, cognitive, and physiological mechanisms, several of which are discussed in greater detail in the section on shared mechanisms.

Longitudinal evidence strengthens the temporal inference. In the Shandong Adolescent Behavior and Health Cohort (approximately 6,900 participants), baseline nightmare frequency and distress predicted suicidal ideation, plans, and attempts over one year, with depressive symptoms acting as a partial mediator [25]. In psychiatric inpatient samples, persistence of nightmares across the initial treatment phase predicted suicide attempts during the ensuing two years, suggesting that chronicity, rather than frequency alone, marks elevated long-term risk [24]. Among adults with prior attempts, frequent nightmares tripled the likelihood of reattempt over two years [30], and national-level cohorts have replicated these associations across broader populations [27]. Systematic reviews of longitudinal studies converge on the conclusion that nightmares prospectively predict suicidal outcomes after accounting for baseline psychopathology, indicating a dose-response relation in which greater nightmare frequency and distress confer higher risk [28,29]. Key clinical, longitudinal, and review studies examining the association between nightmares and suicidality are summarized in Table 1.

**Table 1:** Key clinical and longitudinal studies evaluating the association between nightmares and suicidality. PTSD: post-traumatic stress disorder

Studies	Population	Study design	Key findings
Nadorff et al. (2016) [23]	Adults	Review/conceptual analysis	Proposed nightmares as an independent contributor to suicide risk through emotional dysregulation and sleep disruption.
Littlewood et al. (2016) [24]	Patients with PTSD	Observational study	Nightmares were associated with suicidality through defeat, entrapment, and hopelessness.
Liu et al. (2021) [25]	Adolescents	Longitudinal cohort study	Nightmare frequency and distress predicted future suicidal ideation, plans, and attempts.
Guo et al. (2024) [26]	Young adults	Mediation analysis	Nightmares mediated the relationship between insomnia and suicidal ideation.
Sandman et al. (2017) [27]	General population and veterans	Longitudinal study	Persistent nightmares predicted future suicide risk.
Andrews and Hanna (2020) [28]	Review of psychological studies	Narrative review	Identified emotional dysregulation, fear conditioning, and hopelessness as mediators.
Liu et al. (2020) [29]	Multiple populations	Systematic review and meta-analysis	Nightmares prospectively predicted suicidal outcomes independent of psychiatric comorbidity.
Sjöström et al. (2007) [30]	Suicide attempters	Cohort study	Frequent nightmares were associated with increased risk of repeat suicide attempts.

**Figure 2:**
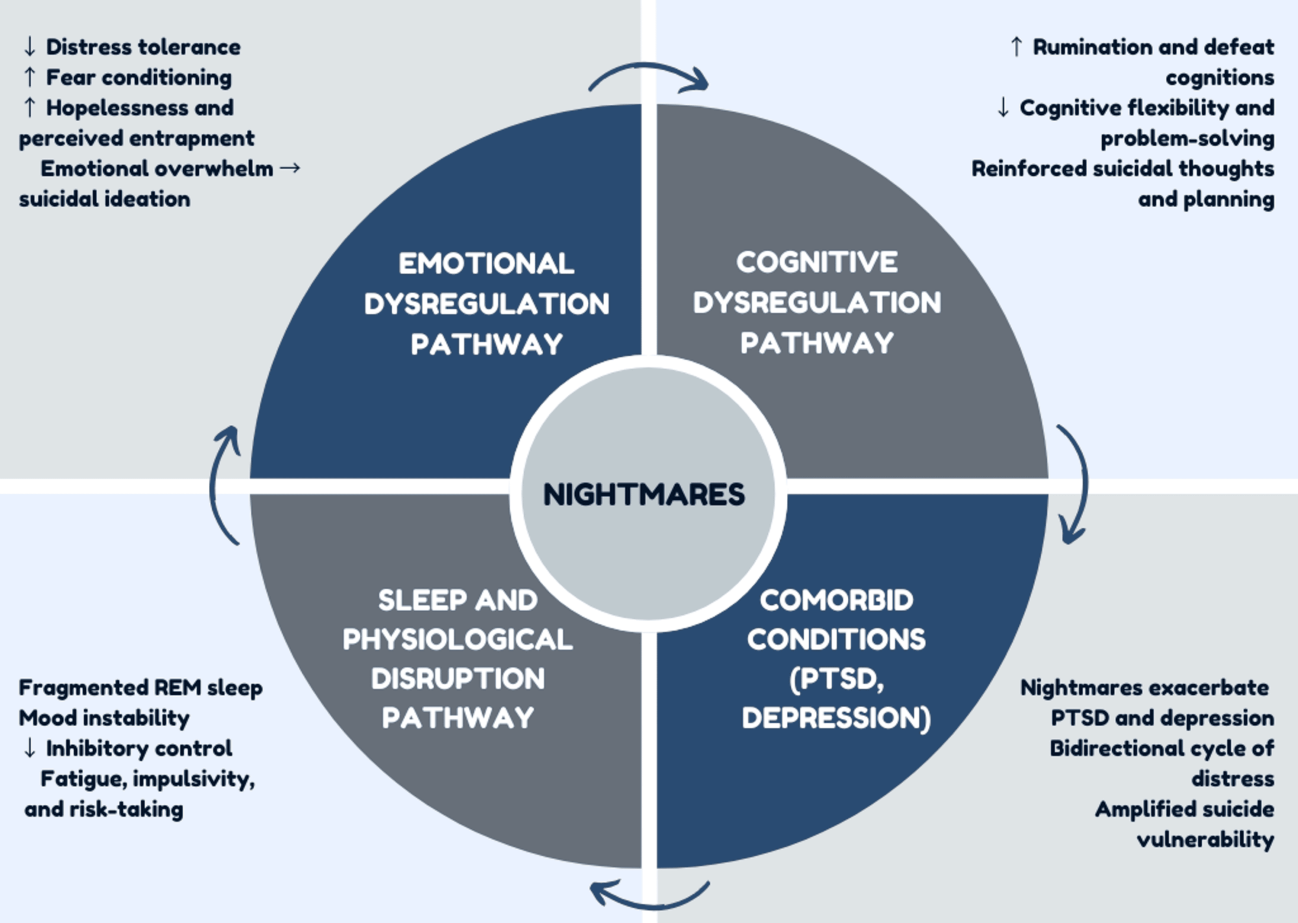
Conceptual framework - nightmares as independent predictors of suicide risk. The diagram depicts three interconnected pathways - emotional dysregulation (reduced distress tolerance, hopelessness), cognitive dysregulation (rumination, negative bias, impaired problem-solving), and sleep/physiological disruption (fear conditioning, hyperarousal, sleep fragmentation) - through which nightmares may increase suicidality [23-25,27-29]. PTSD: post-traumatic stress disorder; REM: rapid eye movement This image was created by the author (Omofolarin Debellotte) of this study using BioRender.com (Toronto, ON: Science Suite Inc.), and no AI assistance was used in its creation.

Clinically, these findings support routine assessment of nightmare frequency and nightmare-related distress within suicide risk evaluations, particularly in patients with PTSD, depressive disorders, or trauma histories. Targeted interventions such as imagery rehearsal therapy can be combined with cognitive behavioral therapy for insomnia and trauma-focused treatments to address both nocturnal symptoms and daytime consequences, with the aim of reducing distress, improving sleep continuity, and attenuating suicidal ideation [24]. Figure 2 provides a conceptual framework that integrates these pathways and highlights potential therapeutic leverage points.

Circadian disruption and suicide

Circadian Rhythm and Mood Regulation

Circadian timing, orchestrated by the suprachiasmatic nucleus (SCN) and its downstream hormonal and neural outputs, exerts pervasive effects on emotion regulation, stress responsivity, and reward processing; misalignment of internal rhythms with the sleep-wake schedule is associated with heightened negative affect, greater stress reactivity, and elevated suicidality [31]. Experimental and translational work indicates that disturbed sleep-circadian organization compromises overnight emotional memory processing and daytime cognitive control, thereby amplifying hopelessness and affective lability-proximal drivers of suicidal ideation and behavior [13,31]. Dysregulated hypothalamic-pituitary-adrenal (HPA) signaling and altered melatonin rhythms frequently accompany circadian misalignment, further destabilizing mood and impulse control in vulnerable individuals [32].

Genetic and Molecular Links (Clock Genes, Melatonin, Lithium Response)

At the molecular level, transcription-translation feedback loops governed by core clock genes (for example, CLOCK, BMAL1, PER/CRY) generate 24-h rhythms in neurons and glia that modulate synaptic signaling, neuroinflammation, and monoaminergic tone relevant to mood and suicidality [33]. Astrocytes are integral to these rhythms; alterations in astrocytic clock function can impair neurotransmitter clearance and glial-neuronal coupling, mechanisms implicated in major depressive and bipolar disorders - conditions carrying high suicide risk [34]. Converging data suggest that interindividual variation in circadian properties has therapeutic implications as follows: greater lithium responsiveness has been observed in patients whose cellular models exhibit enhanced circadian rhythm amplitude and shorter period with lithium exposure, underscoring the bidirectional links between clock function and mood stabilization [31].

Taken together, circadian gene-glia interactions and melatonin/HPA outputs form a biologically plausible pathway through which misalignment may potentiate suicidal thoughts and behaviors [31,34]. Interestingly, lithium, a well-established mood stabilizer with demonstrated anti-suicidal (suicidolytic) properties in bipolar disorder, also exerts important chronobiological effects by modulating circadian rhythm amplitude and clock gene function [34]. This overlap suggests that part of lithium's protective effect against suicidal behavior may be mediated through restoration of circadian homeostasis in addition to its mood-stabilizing actions, highlighting the translational relevance of circadian biology in suicide prevention.

Shift Work, Jet Lag, and Social Jet Lag

**Figure 3:**
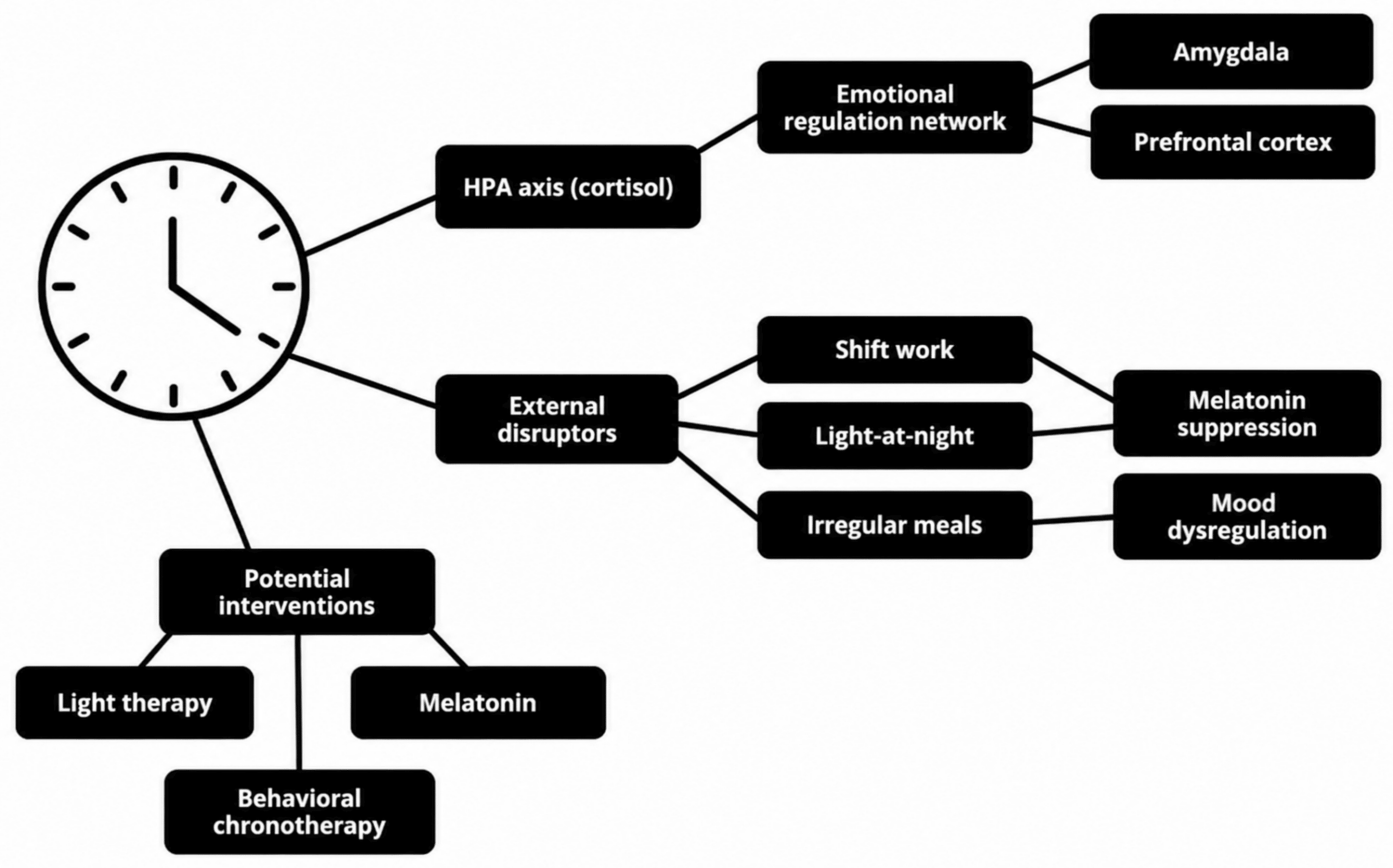
Circadian misalignment and suicide risk. Schematic representation of how disruption of the circadian rhythm interacts with neuroendocrine (HPA axis, cortisol), emotional regulation (amygdala, prefrontal cortex), and external behavioral factors (shift work, light-at-night, irregular meals) to influence suicidal vulnerability. Potential interventions, such as light therapy, melatonin, and behavioral chronotherapy, are presented as modulatory strategies. HPA: hypothalamic-pituitary-adrenal This image was created by the author (Omofolarin Debellotte) of this study using BioRender.com (Toronto, ON: Science Suite Inc.), and no AI assistance was used in its creation.

Environmental desynchronization (night-shift work, rotating schedules, and rapid time-zone travel) disrupts the light-dark-entrained circadian system, shortens and fragments sleep, and is associated with higher rates of depressive symptoms and suicidal ideation in occupational and population studies [35]. Mechanistically, attenuated daytime light exposure, nocturnal light at biologically inappropriate times, and irregular feeding schedules can dampen circadian amplitude, blunt melatonin secretion, perturb serotonin signaling, and destabilize mood regulation [36,37]. Emerging evidence suggests that daytime eating during night shifts, strategic light management, and sleep scheduling may partially re-anchor circadian phase and improve mood, although definitive suicidality endpoints remain limited [38]. More broadly, these data support the hypothesis that chronic misalignment acts as a risk amplifier when layered onto pre-existing psychiatric or psychosocial vulnerabilities. The multidimensional pathways through which circadian misalignment influences suicidal risk, including neuroendocrine, behavioral, and environmental mechanisms, are summarized in Figure 3.

Chronotype and Suicidality

Evening chronotype is consistently linked with shorter sleep duration, greater insomnia severity, increased impulsivity, and higher hopelessness, features closely tied to suicidal risk, particularly in adolescents and young adults [39]. Developmental changes in cortical and white-matter maturation during adolescence are biologically biased toward later timing, while early school start times and social constraints produce “social jet lag,” a chronic misalignment between intrinsic circadian phase and required schedules which is associated with mood instability and self-harm risk [40]. In this context, insomnia symptoms frequently mediate the relationship between eveningness and suicidal ideation, highlighting sleep continuity as a tractable intervention point within circadian-informed care pathways [39].

Clinical Implications and Translational Considerations

From a translational standpoint, integrating circadian assessment into suicide risk evaluation is warranted as follows: brief screening for sleep timing, regularity, and eveningness; inquiry about shift work or frequent travel; and consideration of melatonin timing and light exposure patterns can identify modifiable contributors to risk [41]. Stabilization strategies, morning bright-light therapy, evening light minimization, appropriately timed melatonin, structured sleep-wake schedules, and behavioral chronotherapy represent pragmatic levers to realign phase, improve sleep continuity, and potentially reduce suicidal ideation when combined with evidence-based psychiatric care [42]. In patients with bipolar disorder, collaborative care that attends to circadian regularity and considers lithium’s chronobiological effects may be particularly relevant; in shift-working populations or those with pronounced eveningness, targeted circadian behavioral interventions may complement standard treatments to mitigate risk [31,34,35].

Shared mechanisms underlying sleep disorders and suicide

Neurobiological Pathways

Converging evidence implicates a shared biological axis involving inflammation, hypothalamic-pituitary-adrenal (HPA) dysregulation, and serotonergic imbalance in mediating the association between sleep disturbance and suicidality. Chronic sleep loss heightens systemic inflammation through increased interleukin-6 (IL-6), tumor necrosis factor-α (TNF-α), and interleukin-1β (IL-1β), cytokines that are consistently elevated in both patients with insomnia and individuals who have died by suicide [14,43]. These pro-inflammatory mediators activate the HPA axis, stimulating the release of corticotropin-releasing hormone (CRH) and arginine vasopressin (AVP). Sustained HPA activation weakens negative feedback regulation by downregulating glucocorticoid receptors and upregulating pro-opiomelanocortin (POMC) expression, leading to chronic hypercortisolemia and stress hypersensitivity [44]. Genetic variations such as FKBP5 polymorphisms further reduce glucocorticoid receptor sensitivity, thereby promoting emotional dysregulation and impulsive behaviors that increase suicide vulnerability [44].

In parallel, neuroinflammatory activation alters tryptophan (TRP) metabolism via the kynurenine pathway, diverting TRP away from serotonin synthesis toward the production of neurotoxic metabolites such as quinolinic acid [45]. This metabolic shift contributes to diminished serotonergic tone, impaired mood regulation, and heightened impulsivity, core components of suicidal behavior. Together, these interconnected neuroendocrine and immune processes suggest that sleep disruption amplifies biological stress reactivity while eroding neurochemical resilience, forming a critical pathway to suicidality. Circadian misalignment mechanisms discussed earlier further interact with these inflammatory and serotonergic systems, compounding the physiological vulnerability to suicidal thoughts and behaviors.

Cognitive-Emotional Mechanisms

Sleep disruption also impairs higher-order cognitive control and emotional regulation, creating a psychological environment conducive to suicidal ideation. Individuals with poor sleep exhibit reduced prefrontal inhibition of limbic emotional responses, leading to increased irritability, cognitive rigidity, and maladaptive rumination [46,47]. Rumination, characterized by repetitive, negative thought patterns, has been shown to mediate the relationship between insomnia and suicidal ideation, particularly when emotional regulation capacity is diminished [46]. Sleep deprivation also impairs problem-solving and decision-making abilities, limiting individuals’ capacity to generate adaptive solutions in distress [47]. Meta-analyses demonstrate that suicide attempters, especially those using violent means, show impaired executive function and risky decision-making patterns consistent with sleep-related prefrontal dysfunction [48].

**Figure 4:**
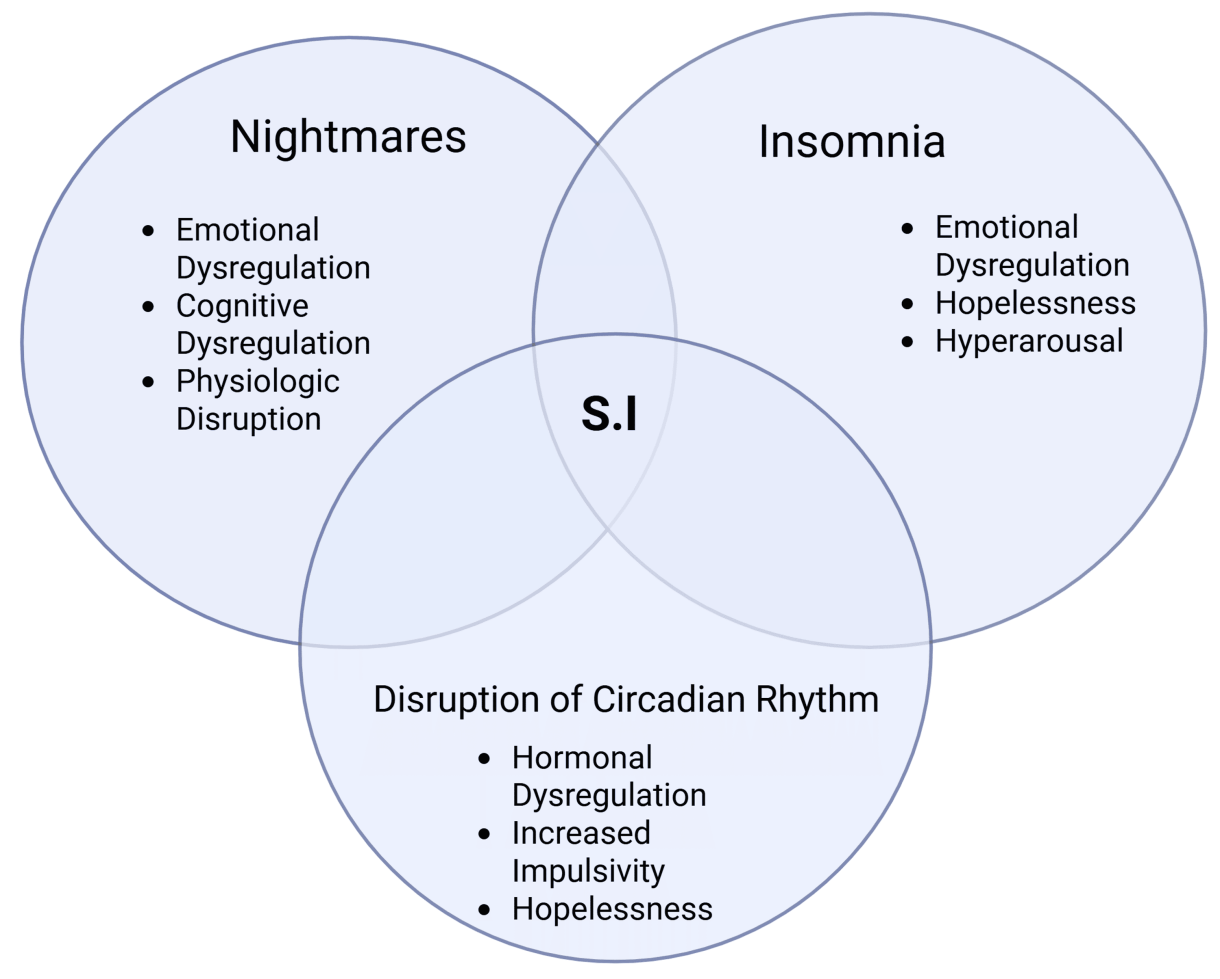
Interrelationships among sleep disturbances and suicidal ideation. This image (Venn diagram) illustrates the interrelationships among nightmares, insomnia, and circadian rhythm disruption, and how they collectively contribute to suicidal ideation (SI) through overlapping mechanisms of emotional, cognitive, and physiological dysregulation. This image was created by the author (Pranati Gudipati) of this study using BioRender.com (Toronto, ON: Science Suite Inc.), and no AI assistance was used in its creation.

Neurobiologically, chronic inflammation and HPA overactivation further dysregulate prefrontal-limbic circuitry, intensifying negative affective bias and emotional dyscontrol. Consequently, insomnia and fragmented sleep bridge the biological and cognitive domains, sustaining hyperarousal while weakening distress tolerance and cognitive flexibility, which together heighten suicide risk. The overlapping mechanisms linking nightmares, insomnia, and circadian disruption to suicidal ideation are summarized in Figure 4.

Interaction With Psychiatric Comorbidities

Psychiatric disorders such as major depression, bipolar disorder, post-traumatic stress disorder (PTSD), and substance use disorders amplify the impact of sleep dysregulation on suicide risk through shared neurobiological substrates. In bipolar disorder, circadian misalignment disrupts mood stabilization and increases impulsivity, reinforcing the sleep-suicide association [49]. In PTSD, recurrent nightmares and hyperarousal maintain chronic sympathetic activation, which sustains suicidal ideation even after controlling for overall sleep quality [50,51]. In alcohol use disorder, insomnia serves as a mediating pathway between alcohol consumption and suicide risk, indicating that addressing sleep disturbances may attenuate suicidality in this population [52].

Collectively, these findings suggest that sleep dysregulation acts as both a symptom and an amplifier of psychiatric comorbidity, bridging neurobiological vulnerability and maladaptive cognition. Addressing sleep disturbances across psychiatric conditions, through cognitive behavioral therapy for insomnia, circadian realignment strategies, and inflammation-modulating approaches, may therefore reduce suicide risk more effectively than treating psychiatric symptoms in isolation.

Given these intertwined biological, cognitive, and psychosocial mechanisms, sleep represents a modifiable target within comprehensive suicide prevention frameworks. Interventions that restore sleep integrity and circadian homeostasis hold promise for mitigating both biological stress sensitivity and cognitive-emotional dysregulation that drive suicidal behavior.

Clinical and therapeutic implications

Screening for Sleep Disturbances in Suicide Risk Assessment

Retrospective and prospective studies provide compelling evidence that disrupted sleep-particularly short sleep duration and insomnia-precedes suicidal behavior. Rolling et al. reported that adolescents who slept 6 h or less on school nights had a significantly greater likelihood of attempting suicide compared with peers achieving 9 h of sleep, emphasizing reduced total sleep time and prolonged latency as key differentiators between suicidal and non-suicidal individuals [53]. Similarly, a Swedish prospective cohort study using causal mediation analysis found that insomnia in the context of short sleep exerted both direct and indirect effects on suicide risk [54]. Beyond insomnia, circadian dysregulation, manifesting as eveningness and reduced rhythmicity, has also been independently associated with suicidal ideation, underscoring the importance of assessing broader sleep-wake disturbances in clinical evaluations [55].

Incorporating structured sleep assessment into suicide risk evaluations using validated tools such as the Insomnia Severity Index (ISI), Pittsburgh Sleep Quality Index (PSQI), or sleep diaries can help identify modifiable contributors to suicidal ideation that may otherwise remain unrecognized. Routine inclusion of these assessments within psychiatric and primary care settings enables early detection and facilitates targeted interventions addressing both sleep and mood regulation.

Therapeutic Interventions: CBT-I, Pharmacotherapy, and Chronotherapy

Cognitive behavioral therapy for insomnia (CBT-I) remains the most evidence-based non-pharmacological intervention for sleep-related suicidality. Randomized controlled trials demonstrate that CBT-I indirectly reduces suicidal ideation through improved sleep quality, emotional regulation, and mood stabilization. Stanley et al. showed that cognitive behavioral therapy for suicide prevention (CBT-SP) was feasible and effective in reducing suicidal ideation [56], while Chen et al. reported that app-based CBT-I not only improved sleep but also reduced the onset of major depressive disorder in youth with insomnia and subclinical depression [57]. Collectively, these findings position CBT-I as the first-line therapeutic approach for individuals with insomnia and suicidal risk.

Pharmacotherapy serves as a short-term adjunct for acute insomnia. Sedative-hypnotics such as zolpidem and midazolam have been shown to alleviate sleep disturbance and modestly reduce suicidal ideation, though long-term use carries risks of dependency and rebound insomnia. A systematic review indicated that alprazolam, zolpidem, and midazolam were associated with a lower risk of suicidal ideation compared with lorazepam and clonazepam, suggesting differential safety profiles among hypnotic agents [58]. Clinicians should weigh these benefits against potential adverse outcomes, ensuring close monitoring and short treatment duration.

Chronotherapy, including controlled light exposure, sleep deprivation therapy, and lithium augmentation, has emerged as a rapid-acting strategy for mood and circadian realignment in treatment-resistant or bipolar depression. Chronotherapeutic approaches such as light therapy and wake therapy can yield swift reductions in suicidal symptoms by stabilizing circadian phase and enhancing mood regulation [59]. More recently, the transdiagnostic sleep and circadian (TranS-C) intervention has demonstrated efficacy in midlife and older adults with serious mental illness, integrating CBT-I principles, circadian stabilization, and emotion regulation techniques [60].

Integration of sleep-focused interventions into suicide prevention strategies

Across interventions, improvement in insomnia symptoms consistently correlates with reductions in suicidal ideation, even when depressive symptom scores remain unchanged. Table 2 summarizes the comparative effectiveness of major interventions.

**Table 2:** Integration of sleep-focused interventions into suicide prevention strategies. TranS-C: transdiagnostic sleep and circadian; CBT-I: cognitive behavioral therapy for insomnia

Intervention	Mechanism	Effect on suicidal ideation	Effect on insomnia
CBT-I [56,57]	Behavioral regulation and cognitive restructuring	Highly effective	Highly effective
Sedative-hypnotics [58]	GABAergic activation	Moderately effective	Highly effective
Chronotherapy [59]	Resetting circadian phase via light or behavioral cues	Moderately effective	Highly effective
TranS-C [60]	Integration of CBT-I, chronotherapy, and emotion regulation	Moderately effective	Moderately effective

Clinically, CBT-I should be considered the first-line intervention, sedative-hypnotics as short-term adjuncts, chronotherapy as a rapid modulation strategy, and TranS-C as a comprehensive transdiagnostic approach for individuals with comorbid mental illness or refractory insomnia.

Given the modifiable nature of sleep disturbances, suicide prevention frameworks should routinely incorporate sleep-specific screening and therapy. Collaboration among psychiatrists, psychologists, and sleep medicine specialists can enhance early detection, personalize intervention, and sustain long-term suicide risk reduction. Addressing sleep dysfunction not only improves rest and circadian stability but also strengthens emotional resilience, cognitive flexibility, and treatment adherence, key pillars of suicide prevention.

Future directions and research gaps

Despite significant advances, the understanding of how sleep disturbances-particularly insomnia, circadian rhythm disruption, and nightmares-contribute to suicidality remains incomplete. Current evidence is largely derived from cross-sectional and randomized controlled studies, which, while informative, limit causal inference and generalizability across diverse populations. Methodological limitations such as recall bias, reverse causation, and inadequate temporal control hinder the ability to delineate whether sleep disorders precipitate suicidal ideation or arise as consequences of it [31,61]. Furthermore, factors such as symptom intensity, coexisting chronic pain, and neurological or psychiatric comorbidities may confound these associations.

Future research should prioritize longitudinal, multi-wave study designs that integrate objective sleep measures, such as actigraphy and polysomnography, with validated suicidality assessments. Such designs would establish temporal precedence and identify mediating mechanisms linking sleep disturbance and suicide risk [4]. Prospective cohorts should also include underrepresented and high-risk populations, including adolescents, shift workers, healthcare professionals, and individuals with chronic medical or psychiatric illnesses. These expansions would enhance external validity and inform the development of population-specific preventive strategies.

Advancing toward a precision psychiatry framework, future studies should incorporate biological, genetic, and circadian markers to improve individual-level risk stratification. Polymorphisms in genes such as CLOCK and FKBP5, inflammatory cytokines (IL-6, TNF-α), and chronotype profiling may together explain interindividual variability in sleep-suicide relationships [1,48]. Integrating these biomarkers into clinical research could facilitate personalized interventions, such as tailored CBT-I protocols, melatonin regulation, or chronotherapy, optimizing treatment efficacy while minimizing overtreatment.

Emerging digital and artificial intelligence (AI) technologies also hold transformative potential. Wearable sleep devices, smartphone-based actigraphy, and AI-driven analytics can continuously monitor sleep-wake cycles, circadian alignment, and mood fluctuations [45]. Predictive algorithms trained on longitudinal datasets could identify high-risk sleep patterns, such as prolonged latency, frequent nocturnal awakenings, or REM fragmentation, and generate automated alerts to support early clinical intervention [62].

Ultimately, combining longitudinal methodologies, biological precision, and digital monitoring will enable the creation of dynamic, real-time suicide risk prediction models that integrate both behavioral and biological signatures. This translational approach may bridge the gap between mechanistic understanding and effective, individualized suicide prevention strategies.

## Conclusions

Sleep disturbances, particularly insomnia, nightmares, and circadian rhythm disruption, emerge as significant, independent, and modifiable risk factors for suicidality. Through converging biological, cognitive, and psychosocial pathways, disordered sleep amplifies emotional dysregulation, hopelessness, and impulsivity, bridging the gap between ideation and suicidal behavior. Evidence from clinical and longitudinal studies indicates that interventions such as CBT-I, imagery rehearsal therapy, and chronotherapeutic approaches consistently improve sleep quality and emotional well-being. Although these interventions may indirectly reduce suicidal ideation by addressing modifiable sleep disturbances, evidence for a direct causal reduction in suicidality remains limited and should be interpreted cautiously. However, the complex interplay between sleep, psychiatric comorbidities, and circadian dysregulation underscores the need for integrated, multidisciplinary prevention models. Incorporating systematic sleep assessment into suicide risk evaluation, supported by emerging digital and circadian-based interventions, offers a pragmatic and scalable approach to prevention. Ultimately, restoring sleep integrity and circadian alignment represents not only a therapeutic target but also a foundational pillar in comprehensive, personalized suicide prevention frameworks.

## References

[1] Hink AB, Killings X, Bhatt A, Ridings LE, Andrews AL (2022). Adolescent suicide - understanding unique risks and opportunities for trauma centers to recognize, intervene, and prevent a leading cause of death. Curr Trauma Rep.

[2] (2025). Suicide data and statistics. https://www.cdc.gov/suicide/facts/index.html.

[3] Bernert RA, Kim JS, Iwata NG, Perlis ML (2015). Sleep disturbances as an evidence-based suicide risk factor. Curr Psychiatry Rep.

[4] Chiang SC, Chen WC, Chou LT (2024). The prospective association between emotional reactivity and adolescent suicidal ideation. Arch Suicide Res.

[5] Lemke T, Hökby S, Carli V, Hadlaczky G (2025). Sleep duration and quality in adolescents: associations with suicidal ideation. J Adolesc.

[6] Baldini V, Gnazzo M, Rapelli G (2024). Association between sleep disturbances and suicidal behavior in adolescents: a systematic review and meta-analysis. Front Psychiatry.

[7] Gowin JL, Stoddard J, Doykos TK, Sammel MD, Bernert RA (2024). Sleep disturbance and subsequent suicidal behaviors in preadolescence. JAMA Netw Open.

[8] Teresi GI, Merranko J, Porta G, Bero K, Poling KD, Brent DA, Goldstein TR (2025). Worsening sleep predicts next-week suicidal ideation in a high-risk adolescent outpatient treatment sample. Suicide Life Threat Behav.

[9] Kivelä LM, van der Does W, Antypa N (2024). Sleep, hopelessness, and suicidal ideation: an ecological momentary assessment and actigraphy study. J Psychiatr Res.

[10] Brüdern J, Hallensleben N, Höller I (2022). Sleep disturbances predict active suicidal ideation the next day: an ecological momentary assessment study. BMC Psychiatry.

[11] Kalmbach DA, Cheng P, Ahmedani BK (2022). Cognitive-behavioral therapy for insomnia prevents and alleviates suicidal ideation: insomnia remission is a suicidolytic mechanism. Sleep.

[12] Meyer N, Lok R, Schmidt C (2024). The sleep-circadian interface: a window into mental disorders. Proc Natl Acad Sci U S A.

[13] Dolsen EA, Prather AA, Lamers F, Penninx BW (2021). Suicidal ideation and suicide attempts: associations with sleep duration, insomnia, and inflammation. Psychol Med.

[14] Kim J, Kwon R, Yun H, Lim GY, Woo KS, Kim I (2024). The association between long working hours, shift work, and suicidal ideation: a systematic review and meta-analyses. Scand J Work Environ Health.

[15] Diefenbach GJ, Lord KA, Stubbing J (2024). Brief cognitive behavioral therapy for suicidal inpatients: a randomized clinical trial. JAMA Psychiatry.

[16] Pigeon WR, Crean HF, Cerulli C, Gallegos AM, Bishop TM, Heffner KL (2022). A randomized clinical trial of cognitive-behavioral therapy for insomnia to augment posttraumatic stress disorder treatment in survivors of interpersonal violence. Psychother Psychosom.

[17] McCall WV, Benca RM, Rosenquist PB (2019). Reducing Suicidal Ideation Through Insomnia Treatment (REST-IT): a randomized clinical trial. Am J Psychiatry.

[18] Nazem S, Sun S, Barnes SM (2024). Impact of an internet-based insomnia intervention on suicidal ideation and associated correlates in veterans at elevated suicide risk. Transl Behav Med.

[19] Batterham PJ, Thorndike FP, Gerwien R, Botbyl J, Ritterband LM, Maricich Y, Christensen H (2024). Sleep-specific outcomes attributable to digitally delivered cognitive behavioral therapy for insomnia in adults with insomnia and depressive symptoms. Behav Sleep Med.

[20] Soltani Z, Parizad N, Radfar M, Alinejad V, Arzanlo M, Haghighi M (2024). The effect of the Yara smartphone application on anxiety, sleep quality, and suicidal thoughts in patients with major depressive disorder in Iran: a randomized controlled trial. BMC Psychiatry.

[21] McCall WV, Black CG (2013). The link between suicide and insomnia: theoretical mechanisms. Curr Psychiatry Rep.

[22] Rogante E, Cifrodelli M, Sarubbi S, Costanza A, Erbuto D, Berardelli I, Pompili M (2024). The role of emotion dysregulation in understanding suicide risk: a systematic review of the literature. Healthcare (Basel).

[23] Nadorff MR, Pearson MD, Golding S (2016). Explaining the relation between nightmares and suicide. J Clin Sleep Med.

[24] Littlewood DL, Gooding PA, Panagioti M, Kyle SD (2016). Nightmares and suicide in posttraumatic stress disorder: the mediating role of defeat, entrapment, and hopelessness. J Clin Sleep Med.

[25] Liu X, Yang Y, Liu ZZ, Jia CX (2021). Longitudinal associations of nightmare frequency and nightmare distress with suicidal behavior in adolescents: mediating role of depressive symptoms. Sleep.

[26] Guo Z, Han X, Kong T, Wu Y, Kang Y, Liu Y, Wang F (2024). The mediation effects of nightmares and depression between insomnia and suicidal ideation in young adults. Sci Rep.

[27] Sandman N, Valli K, Kronholm E, Vartiainen E, Laatikainen T, Paunio T (2017). Nightmares as predictors of suicide: an extension study including war veterans. Sci Rep.

[28] Andrews S, Hanna P (2020). Investigating the psychological mechanisms underlying the relationship between nightmares, suicide and self-harm. Sleep Med Rev.

[29] Liu RT, Steele SJ, Hamilton JL (2020). Sleep and suicide: a systematic review and meta-analysis of longitudinal studies. Clin Psychol Rev.

[30] Sjöström N, Waern M, Hetta J (2007). Nightmares and sleep disturbances in relation to suicidality in suicide attempters. Sleep.

[31] Whiting C, Bellaert N, Deveney C, Tseng WL (2023). Associations between sleep quality and irritability: testing the mediating role of emotion regulation. Pers Individ Dif.

[32] Yin Q, Cheng MY, Wang S (2025). Cervicogenic sleep disorder syndrome: definition and classification recommendations. Transl Perioper Pain Med.

[33] McCarthy MJ, Gottlieb JF, Gonzalez R (2022). Neurobiological and behavioral mechanisms of circadian rhythm disruption in bipolar disorder: a critical multi-disciplinary literature review and agenda for future research from the ISBD task force on chronobiology. Bipolar Disord.

[34] Steardo Jr L, de Filippis R, Carbone EA, Segura-Garcia C, Verkhratsky A, De Fazio P (2019). Sleep disturbance in bipolar disorder: neuroglia and circadian rhythms. Front Psychiatry.

[35] Wei T, Li C, Heng Y (2020). Association between night-shift work and level of melatonin: systematic review and meta-analysis. Sleep Med.

[36] Molcan L, Sutovska H, Okuliarova M, Senko T, Krskova L, Zeman M (2019). Dim light at night attenuates circadian rhythms in the cardiovascular system and suppresses melatonin in rats. Life Sci.

[37] Hu J, Ye H, Wang M (2025). The effects of food on circadian rhythm: a comprehensive review. eFood.

[38] Wright CJ, Milosavljevic S, Pocivavsek A (2023). The stress of losing sleep: sex-specific neurobiological outcomes. Neurobiol Stress.

[39] Bradford DR, Biello SM, Russell K (2021). Insomnia symptoms mediate the association between eveningness and suicidal ideation, defeat, entrapment, and psychological distress in students. Chronobiol Int.

[40] Simon KC, Cadle C, Shuster AE, Malerba P (2025). Sleep across the lifespan: a neurobehavioral perspective. Curr Sleep Med Rep.

[41] Gordijn MC (2022). 33rd annual meeting of the Society for Light Treatment and Biological Rhythms (SLTBR), 23-25 June 2022, Manchester, UK. Clocks Sleep.

[42] Lévi FA, Okyar A, Hadadi E, Innominato PF, Ballesta A (2024). Circadian regulation of drug responses: toward sex-specific and personalized chronotherapy. Annu Rev Pharmacol Toxicol.

[43] Navarro D, Marín-Mayor M, Gasparyan A, García-Gutiérrez MS, Rubio G, Manzanares J (2023). Molecular changes associated with suicide. Int J Mol Sci.

[44] Berardelli I, Serafini G, Cortese N, Fiaschè F, O'Connor RC, Pompili M (2020). The involvement of hypothalamus-pituitary-adrenal (HPA) axis in suicide risk. Brain Sci.

[45] Sarawi WS (2025). Neurochemical insights into the role of tryptophan metabolites and kynurenine pathway in insomnia and its psychological and neurological comorbidities. Mol Neurobiol.

[46] Holdaway AS, Luebbe AM, Becker SP (2018). Rumination in relation to suicide risk, ideation, and attempts: exacerbation by poor sleep quality?. J Affect Disord.

[47] Lim J, Dinges DF (2010). A meta-analysis of the impact of short-term sleep deprivation on cognitive variables. Psychol Bull.

[48] Perrain R, Dardennes R, Jollant F (2021). Risky decision-making in suicide attempters, and the choice of a violent suicidal means: an updated meta-analysis. J Affect Disord.

[49] Tonon AC, Nexha A, da Silva MM, Gomes FA, Hidalgo MP, Frey BN (2024). Sleep and circadian disruption in bipolar disorders: from psychopathology to digital phenotyping in clinical practice. Psychiatry Clin Neurosci.

[50] El-Solh AA (2018). Management of nightmares in patients with posttraumatic stress disorder: current perspectives. Nat Sci Sleep.

[51] Weber FC, Norra C, Wetter TC (2020). Sleep disturbances and suicidality in posttraumatic stress disorder: an overview of the literature. Front Psychiatry.

[52] Nadorff MR, Salem T, Winer ES, Lamis DA, Nazem S, Berman ME (2014). Explaining alcohol use and suicide risk: a moderated mediation model involving insomnia symptoms and gender. J Clin Sleep Med.

[53] Rolling J, Ligier F, Rabot J, Bourgin P, Reynaud E, Schroder CM (2024). Sleep and circadian rhythms in adolescents with attempted suicide. Sci Rep.

[54] Hedström AK, Hössjer O, Bellocco R, Ye W, Trolle LY, Åkerstedt T (2021). Insomnia in the context of short sleep increases suicide risk. Sleep.

[55] Rumble ME, McCall WV, Dickson DA, Krystal AD, Rosenquist PB, Benca RM (2020). An exploratory analysis of the association of circadian rhythm dysregulation and insomnia with suicidal ideation over the course of treatment in individuals with depression, insomnia, and suicidal ideation. J Clin Sleep Med.

[56] Stanley B, Brown G, Brent DA (2009). Cognitive-behavioral therapy for suicide prevention (CBT-SP): treatment model, feasibility, and acceptability. J Am Acad Child Adolesc Psychiatry.

[57] Chen SJ, Que JY, Chan NY (2025). Effectiveness of app-based cognitive behavioral therapy for insomnia on preventing major depressive disorder in youth with insomnia and subclinical depression: a randomized clinical trial. PLoS Med.

[58] Valentino K, Teopiz K, Wong S (2025). A systematic review of anti-suicidal effects of sedative-hypnotics and cognitive behavioral therapy for insomnia. CNS Spectr.

[59] Benedetti F, Riccaboni R, Locatelli C, Poletti S, Dallaspezia S, Colombo C (2014). Rapid treatment response of suicidal symptoms to lithium, sleep deprivation, and light therapy (chronotherapeutics) in drug-resistant bipolar depression. J Clin Psychiatry.

[60] Sarfan LD, Gasperetti CE, Gumport NB, Harvey AG (2022). Outcomes from the transdiagnostic sleep and circadian intervention (TranS-C) for midlife and older adults with serious mental illness and sleep and circadian dysfunction. Behav Ther.

[61] Al-Remawi M, Ali Agha AS, Al-Akayleh F, Aburub F, Abdel-Rahem RA (2024). Artificial intelligence and machine learning techniques for suicide prediction: integrating dietary patterns and environmental contaminants. Heliyon.

[62] Aziz S, Ali AA, Aslam H (2025). Wearable artificial intelligence for sleep disorders: scoping review. J Med Internet Res.

